# Sulfated Galactofucans: An Outstanding Class of Fucoidans with Promising Bioactivities

**DOI:** 10.3390/md20070412

**Published:** 2022-06-24

**Authors:** Ahmed Zayed, Jose Avila-Peltroche, Mona El-Aasr, Roland Ulber

**Affiliations:** 1 Institute of Bioprocess Engineering, Technical University of Kaiserslautern, Gottlieb-Daimler-Straße 49, 67663 Kaiserslautern, Germany; ulber@mv.uni-kl.de; 2 Department of Pharmacognosy, College of Pharmacy, Tanta University, El-Guish Street (Medical Campus), Tanta 31527, Egypt; moelaasar@pharm.tanta.edu.eg; 3 Department of Life Science, Chosun University, Gwangju 61452, Korea; jose_avila22@hotmail.com

**Keywords:** bioactives, brown seaweeds, fucoidans, heteropolysaccharides, structural features, sulfated galactofucans

## Abstract

Fucoidans encompass versatile and heterogeneous sulfated biopolysaccharides of marine origin, specifically brown algae and marine invertebrates. Their chemistry and bioactivities have been extensively investigated in the last few decades. The reported studies revealed diverse chemical skeletons in which l-fucose is the main sugar monomer. However, other sugars, i.e., galactose, mannose, etc., have been identified to be interspersed, forming several heteropolymers, including galactofucans/fucogalactans (G-fucoidans). Particularly, sulfated galactofucans are associated with rich chemistry contributing to more promising bioactivities than fucans and other marine polysaccharides. The previous reports in the last 20 years showed that G-fucoidans derived from *Undaria pinnatifida* were the most studied; 21 bioactivities were investigated, especially antitumor and antiviral activities, and unique biomedical applications compared to other marine polysaccharides were demonstrated. Hence, the current article specifically reviews the biogenic sources, chemistry, and outstanding bioactivities of G-fucoidans providing the opportunity to discover novel drug candidates.

## 1. Introduction

Fucoidans are unique products of marine organisms, specifically sulfated polysaccharides derived from brown algae and marine invertebrates [1]. They have gained great interest in the last few decades from different fields of sciences, including chemistry, biology, medicine, nutrition, and formulations [2,3,4,5]. All this interest is attributed to the diverse physicochemical, chemical, and biological characteristics [6,7]. These characteristics are relatively related to each other and have been studied previously in a wide variety of literature [8,9,10,11,12,13]. Hence, biological investigations are always performed after full chemical and physicochemical characterizations of purified fucoidans [14,15,16,17]. Monosaccharide composition, molecular weight, sulfation pattern, and sulfation content were found to be the most predominant factors that contribute to fucoidans’ molecular mechanisms [11,12,18,19].

The aforementioned characteristics of fucoidans were demonstrated to be highly dependent on many factors, including downstream processes such as extraction either by classical solvent or non-conventional extraction methods [20,21,22], fractionation and purification methods [23,24,25], biogenic sources [24,26,27], and season of harvesting [28]. For instance, fucoidans isolated from sea cucumber showed a homogeneous chemical structure in comparison with brown seaweeds [27]. All of these factors have contributed to the chemical diversity and complexity of fucoidans, the lack of reproducibility of investigational results, and the difficulty of their approval by drug authorities and clear understanding of structure–activity relationships [29].

**Figure 1:**
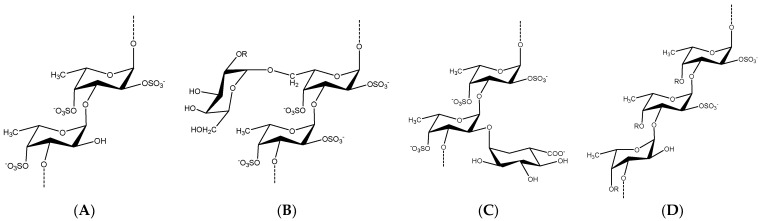
Different chemical backbones of fucoidans isolated from marine seaweeds in which *α*-l-fucopyranosyl residue (Fuc*p*) is the major sugar monomer. (**A**) A sulfated fucan (F-fucoidans) isolated from *Lessonia* sp., where the Fuc*p* monomers are linked by *α*(1→3) and sulfated at *O*-4 and partially at *O*-2 [34]. (**B**) A sulfated galactofucan (G-fucoidans) isolated from *Hormophysa cuneiformis*. *β*-d-Galactopyranosyl residues (Gal*p*) are found mostly at the periphery of molecules as (1→6)-linked (R=H or SO_3_^−^) [36]. (**C**) Fucoidan containing uronic acid at *O*-2 isolated from *Cladosiphon okamuranus* [37]. (**D**) A sulfated xylofucan from *Punctaria plantaginea*. *β*-d-Xylopyranosyl residues (R=H or Xyl*p*) randomly substitute Fuc*p* monomers at *O*-4 [38].

Following the recent advances in chromatographic methods, spectral analysis, and biochemical characterization of many fucoidanases and sulfatases, the native chemical structures of many fucoidans have been elucidated [25,30,31,32]. Hence, identification of fucoidans’ monomeric composition, site of branching, and sulfation pattern has become more feasible and reasonable than before. For instance, a number of sugar monomers were identified, i.e., fucose, galactose, glucose, xylose, mannose, mannitol, and rhamnose, in addition to uronic acids [33]. The chemical diversity of fucoidans has resulted in various backbones that can be classified according to monomeric composition into sulfated fucans (F-fucoidans), galactofucans/fucogalactans (G-fucoidans or G-fucans), fucomannoglucuronans (GA or U-fucoidans), and others [10,34,35,36]. Representative examples are demonstrated in Figure 1.

F-fucoidans are the most simple and regular form of fucoidans, where the fucoidan backbone is composed of α-l-fucose only, i.e., homopolymers of l-fucose. This form is abundantly extracted from marine invertebrates (e.g., sea cucumber) and to a lesser extent from brown algae [39,40]. However, the International Union of Pure and Applied Chemistry (IUPAC) has recently distinguished the sulfated fucans from fucoidans and classified them in a separate class [41]. The second class is the G-fucoidans, which are highly common in brown algae and composed of *β*-d-galactose in addition to the α-l-fucose in variable ratios [37,42]. Based on previously reported findings, several species of Laminariales and Fucales are richer in G-fucoidans. In addition, the third and other classes of fucoidans are highly heterogeneous with a contribution of several other sugar monomers such as glucose, mannose, rhamnose, xylose, and uronic acids [38,43]. It is also noteworthy to mention that various or a mixture of the different classes of fucoidan oligomers may be found in the same organism extracts or fractions [36,44].

Particularly, G-fucoidans have been more investigated recently and showed an outstanding broad spectrum of bioactivities and stability in response to autohydrolysis in comparison with other classes of fucoidans and marine polysaccharides [45,46]. For instance, they showed antioxidant, antiviral, anti-inflammatory, anti-hyperglycemic, anticoagulant, and antitumor activities [47,48,49] and were promising candidates for prevention and treatment of coronavirus disease 2019 (COVID-19) [50]. The current production status of brown seaweeds is continuously increasing [51], specifically for *Saccharina* or *Laminaria* species and *Undaria pinnatifida* which are major G-fucoidan producers. For instance, the annual *Saccharina* production was estimated at 5.7 million tons worth USD 330 million and 2.1 million tons of *U. pinnatifida* worth USD 0.9 billion [52].

Hence, the current review aims at addressing different aspects of G-fucoidans, including biogenic sources, chemistry, and reported bioactivities. Moreover, proposed structure–activity relationships are highlighted. This may help the further investigation and consequences of galactose presence in the fucoidan chemical backbone.

## 2. Occurrence, Distribution, and Chemistry

Brown seaweeds, in contrast to marine invertebrates, can synthesize more complicated, diverse, and heterogeneous fucoidan backbones, including glycosidic linkages, monomeric composition, and branching sites [53,54,55]. Therefore, various G-fucoidans with different fucose:galactose ratios have been reported in the different brown algae orders, including Fucales, Laminariales, and Dictyoales [56,57].

Traces of other sugars may be found, as in the case of *Dictyota menstrualis* [58] and *Sargassum* sp. [59]. Nevertheless, the presence of high percentages of glucose, i.e., fucose:galactose:glucose ratio of 1:0.3:0.25, may indicate contamination of the G-fucoidan with laminarin [60]. In such cases, fucoidans are partially purified by ethanol or cetyltrimethylammonium bromide (CTAB) precipitation and not purified by a specific chromatographic method, including anion exchange resin using diethylaminoethyl cellulose (DEAE-C) [61] or affinity chromatography [62].

In addition, previous studies, with the aid of advanced spectral analyses, i.e., 2D NMR (e.g., HMQC, TOCSY, and NOESY) and mass spectrometry, have attempted to reveal many structural features of G-fucoidans of various biogenic sources, including glycosidic linkages, sugar configuration, branching sites, sulfation pattern, and galactose position [6,63,64]. In addition, they could deduce tentative structure bioactivity relationships, as in the case of the anti-inflammatory mechanism of galactofucan isolated from *Saccharina japonica* [65].

**Figure 2:**
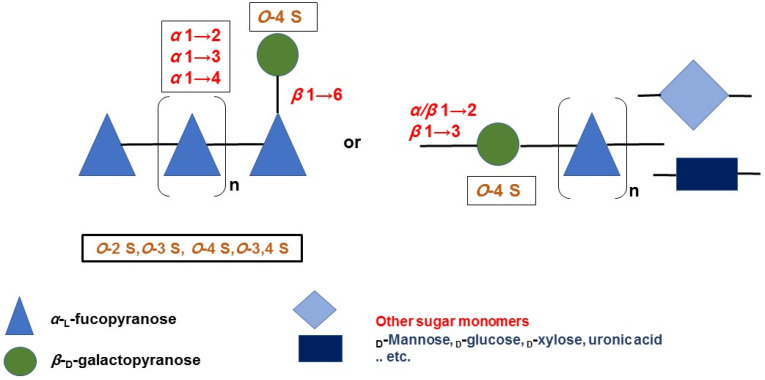
Structure models of sulfated galactofucans isolated from brown algae showing several possibilities of glycosidic linkages and sulfation patterns.

The results of spectral analyses showed that *α*-l-fucopyranose (Fuc*p*) and *β*-d-galactopyranose (Gal*p*) are identified mainly, in which Fuc*p* forms the major backbone and is linked via (1→4) and/or (1→3), while the *β*-d-galactopyranose molecules are found at branching sites, usually at (1→6), as in case of the G-fucoidan isolated from *Hormophysa cuneiformis*. In addition, the sulfation pattern is variable based on the glycosidic linkages. For instance, sulfate groups may occupy 2-*O* and 4-*O* in →3Fuc*p*1→ or 2-*O* and 3-*O* in →4Fuc*p*1→, in addition to 2-*O* in →3,4Fuc*p*1→ [36]. Other models of sulfated galactofucans derived from *Sargassum thunbergii* were found to possess →3Fuc*p*1→ as a main backbone with a 2-*O*-sulfated and 2,4-*O*-disulfated pattern, while the Gal*p* residues interspersed Fuc*p* in the main chain were linked mainly with →6Gal*p*1→ and 4-*O* sulfation [46]. Moreover, G-fucoidan isolated from *S. polycystum* was built up mainly of a 4-*O* sulfated →3Fuc*p*1→ backbone containing single →2Gal*p*1→ residues sulfated similarly at the 4-*O* position [66]. Several other models are demonstrated in Figure 2 and Table 1 and in relation to their biomedical applications.

## 3. Potential Pharmacological Activities

Diversity in fucoidans’ chemical structures is always associated with promising and various bioactivities, which are typical with G-fucoidans [11,103]. The following subsection highlight these bioactivities. A systematic review in the Google Scholar and Scopus databases was performed using the keywords “galactofucan*”, “bioactivity”, and “biomedical”. A timeframe was not pre-established; however, the selection criteria were set to include full-length English articles in peer-reviewed journals and articles addressing biological activities along with the chemical properties of galactofucans. Moreover, publications in which the chemical compositions or structures based on previous analyses were consulted in addition to these original articles. Nevertheless, they were not included in the current review if they did not include any biological assessment. We also identified additional sources through manual reference tracing within the selected articles.

**Figure 3:**
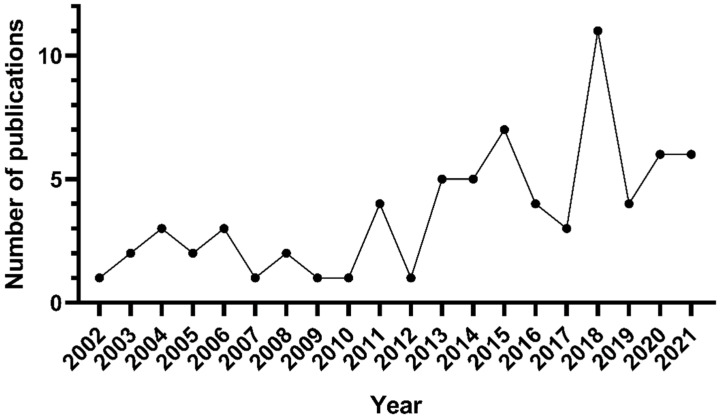
Number of publications on the bioactivity of galactofucans by year between 2002 and 2021.

Seventy-two articles that evaluated the various bioactivities of galactofucans from brown seaweeds were identified between 2002 and 2021. In general, research on this topic went from 1.77 papers year^−1^ during 2002–2010 to 5 papers year^−1^ for the most recent period (2011–2021). These values show increasing attention to galactofucan bioactivities during recent years (Figure 3).

**Figure 4:**
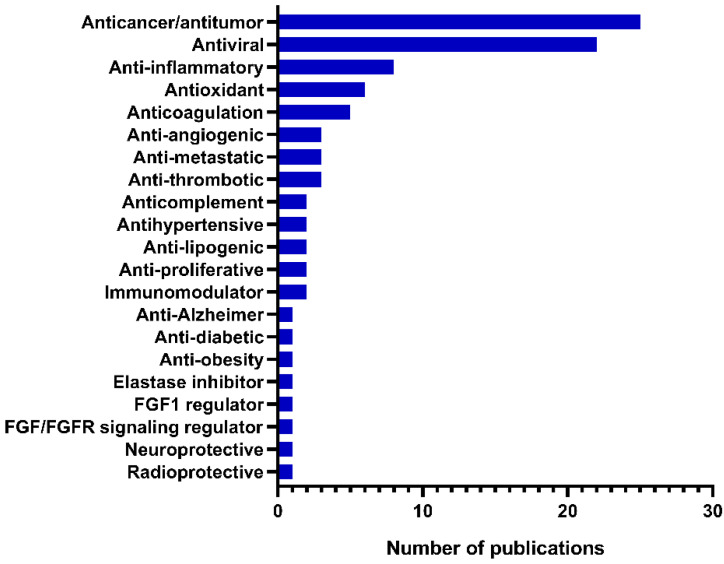
Number of publications on bioactivity of galactofucans by reported biological property between 2002 and 2021.

Among the reported pharmacological applications, antitumor/anticancer and antiviral activities were by far the most studied, with 25 and 22 publications, respectively (Figure 4).

**Figure 5:**
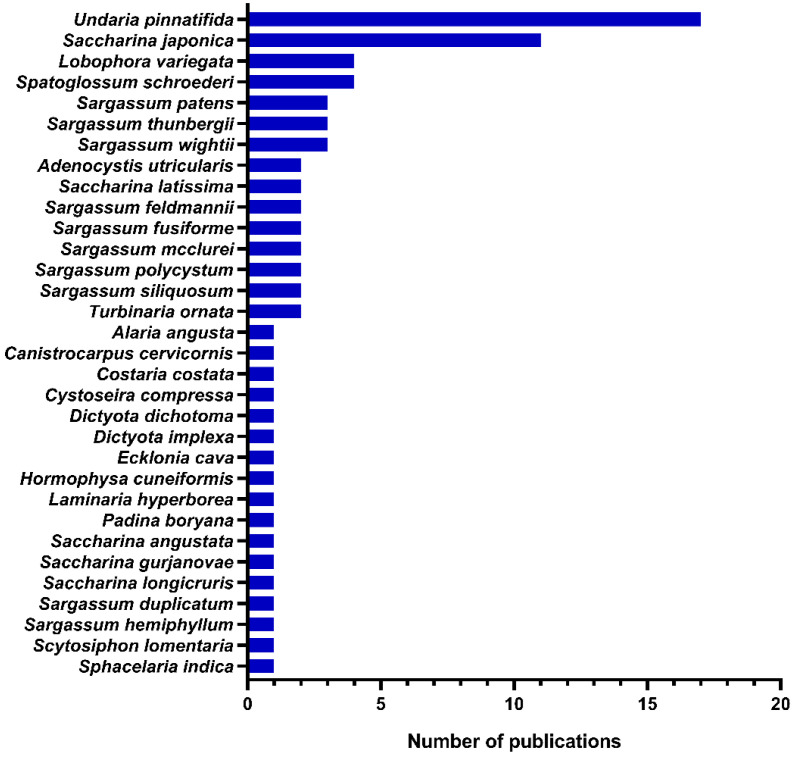
Number of publications on bioactivity of galactofucans by species between 2002 and 2021.

Our literature survey revealed that galactofucans from 31 brown algal species had shown promising biological activities. *Undaria pinnatifida* and *Saccharina japonica* were the most studied species with 17 and 11 publications, respectively. It is also worth noting that the genera *Sargassum* (10 species) and *Saccharina* (5 species) presented the highest numbers of species (Figure 5). Special attention has been paid to sporophylls of *U. pinnatifida* (*mekabu*), which is used in Japan and Korean cuisines, as well as in other countries of East Asia [125,126]. *Mekabu*’s fucoidan is a galactofucan with anticancer/antitumor, anticoagulation, antimetastatic, antioxidant, antithrombotic, and antiviral activities [122]. Currently, the company Biocorp, a Korean manufacturer of health food made of naturally derived ingredients, sells *mekabu*’s fucoidan from Jeollanam-do Sea in South Korea [127].

**Figure 6:**
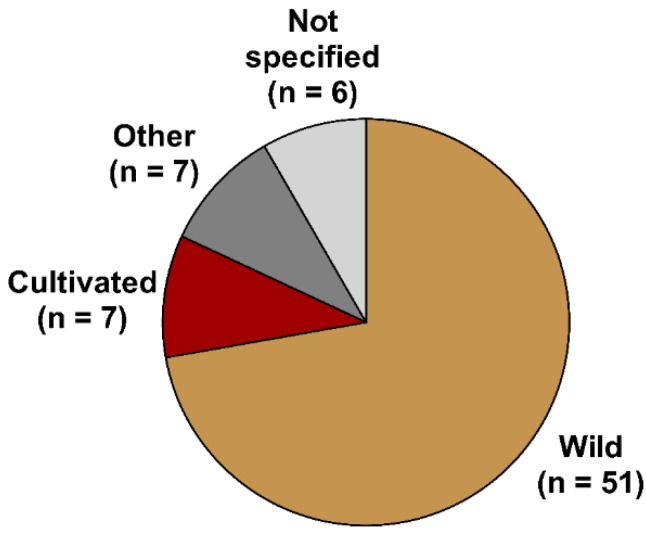
Number of publications (*n*) classified according to the source of seaweed biomass used for extracting galactofucans with reported bioactivities.

The majority of the publications used wild seaweeds for galactofucan extraction. A low number utilized cultivated biomass from open-sea farms, corresponding to either *U. pinnatifida* (and more specifically the sporophylls) or *S. japonica* (Figure 6). Standardizing bioactivity using seaweed cultivars is necessary if this industry wants to develop high-value markets for functional foods, cosmeceuticals, nutraceuticals, and pharmaceuticals [128]. In the case of G-fucoidans, this would imply more studies using cultivated species to identify cultivars with high bioactivities.

**Figure 7:**
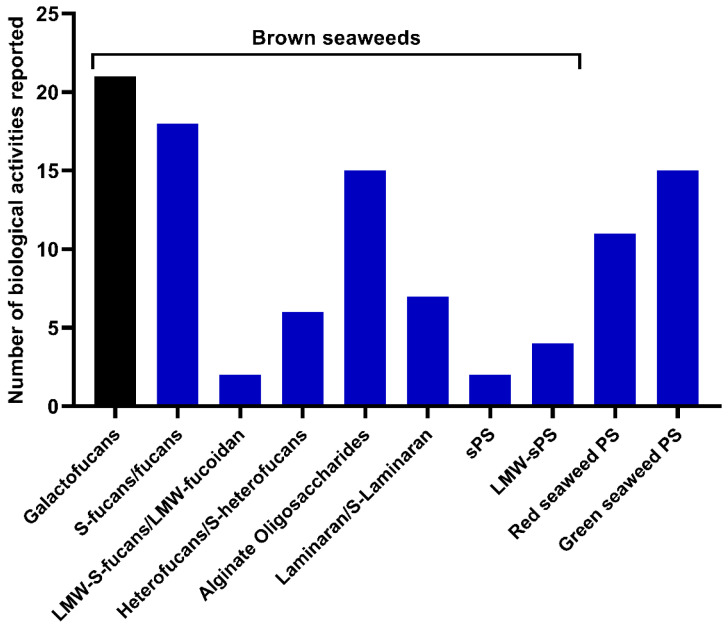
Comparison of biological properties of galactofucans (black) reviewed in this study and others reported for polysaccharides (PS) from marine macroalgae (blue) [45,129].

Bioactivities were mostly evaluated using in vitro tests in most of the publications evaluated (*n* = 58). Studies involving both in vitro and in vivo (*n* = 8) and only in vivo tests (*n* = 5) accounted for fewer publications. Galactofucans showed the highest number of reported bioactivities compared to other classes of polysaccharides in brown, red, and green seaweeds (Figure 7). These findings might support the idea that, among fucoidans, the galactofucan components concentrate the biological activities [24].

### 3.1. Anticancer/Antitumor Activity

Several studies have reported the anticancer/antitumor activities of galactofucans in different cancer cell lines, as well as antiproliferative, antimetastasis, and antiangiogenic effects. For example, *mekabu*’s galactofucans showed antitumor activity against PC-3 (prostate cancer), HeLa (cervical cancer), A549 (alveolar carcinoma), HepG2 (hepatocellular carcinoma), MCF-7 (breast adenocarcinoma), and A-549 (lung carcinoma) cells, in a similar or superior pattern to a commercial fucoidan from *Fucus vesiculosus*. Structural elucidation of this fucoidan demonstrated an *O*-acetylated sulfated galactofucan backbone with a fucose:galactose ratio of 1.0:1.1, sulfate (0.97 mol/mol), and acetate (0.24 mol/mol), in addition to the absence of uronic acids. However, this research did not determine any chemical property of the control fucoidan used, i.e., commercial fucoidan from *F. vesiculosus*, and hence, comparison based on structure–activity relationship was not possible [44,117,130], (Table 2). Another study using Hca-F (mouse hepatocarcinoma) cells showed that the anticancer activity of *mekabu* might be mediated through the mechanism involving inactivation of the NF-κB pathway mediated by PI3K/Akt and ERK signaling pathways [123]. In addition, *Saccharina latissima* has been proposed as a more appropriate source of sulfated galactofucan with antitumor activity superior to commercial heparins (Table 2). When compared to *F. vesiculosus* or other algae species, *S. latissima* showed lower contents of co-extractable compounds (e.g., phenolic derivatives), a requirement for any potential medical application [107].

Despite their promising bioactivities, fucoidans’ high molecular weight and viscous nature (including galactofucans) may limit their use as therapeutic agents. In this regard, lower-sized fucoidans have emerged as a possible solution to these problems. For example, low-molecular-weight (LMG) mannogalactofucans derived from G-fucoidans of *mekabu* strongly attenuated the growth of human prostate cancer cells both in vitro and in vivo [116]. Similarly, an LMG sulfated galactofucan from *Sargassum thunbergii*, with high fucose content, presented better antitumor and antiangiogenic effects against human lung cancer A549 and human umbilical vein endothelial cells, respectively [63].

Our bibliographic search revealed that most of the studies have focused on analyzing the anticancer effect in vitro. So far, only the galactofucans from *U. pinnatifida* and *Sargassum thunbergii* have shown promising activities against prostate and lung cancer in vivo, respectively. In both cases, a xenografted mouse model was used to study tumor inhibition [46,116].

### 3.2. Antiviral Activity

Galactofucans show antiviral properties against a number of highly pathogenic viruses, including the human immunodeficiency virus (HIV-1) (Table 3). They can block the early steps of HIV entry into target cells [84] or inhibit reverse transcriptases [131]. According to [100], the inhibitory activity of fucoidans is specific against viruses that use heparan sulfate as the primary cell receptor. Although all fucans can be considered as potential anti-HIV agents, it seems that galactofucans are the most effective inhibitors among them. Studies have also shown that G-fucoidans present high and selective antiviral activity against herpes simplex virus type 1 and 2 (HSV-1 and HSV-2), showing 50% cytotoxic concentration (CC_50_) >1000 μg/mL against Vero B cells and IC_50_ values in the range 0.7–10.0 μg/mL [78], including both acyclovir (ACV)-sensitive and -resistant strains [85,86,87]. In addition, sulfated xylogalactofucan (F2S2) from *Saccharina angustata* inhibited the HSV-1 adsorption/attachment to cells with higher potency (0.65 μg/mL) and selectivity index (SI > 1538) than sulfated alginate (0.2–25 μg/mL) from the same species [97]. Moreover, the antiherpetic effect of a commercial sulfated galactofucan from *U. pinnatifida* has been confirmed in vivo [118]. All these results highlight the importance of the sulfated galactofucans for the prevention of herpetic infections.

Interestingly, this antiherpetic effect might help in the treatment of Alzheimer’s disease (AD) patients. A galactofucan from *U. pinnatifida* prevented the HSV-1-induced accumulation of the characteristic abnormal molecules of AD brains, Aβ and P-tau [111]. Other studies have also demonstrated the antiviral properties of these macromolecules against avian influenza A viruses, Coxsackie virus, and human cytomegalovirus [68,121,122]. Overall, only five brown algal species, i.e., *Adenocystis utricularis*, *Dictyota dichotoma*, *Sargassum patens*, *Sphacelaria indica*, and *U. pinnatifida*, have shown similar or superior antiviral activities against HIV-1, HSV-1, HSV-2, and/or CVB3 when compared to standard antiviral drugs (Table 3).

Galactofucans might also be good candidates for preventing and/or treating severe acute respiratory syndrome coronavirus 2 (SARS-CoV-2), the virus responsible for the current COVID-19 pandemic. A recent study showed that G-fucoidans from *S. japonica* presented a strong bind ability to the virus spike glycoproteins (SGPs), one of the targets for COVID treatment [50]. Furthermore, an in silico study revealed that sulfated galactofucan achieved stable binding with receptor-binding domain (RBD) of SARS CoV-2’s spike protein (S-protein) at two sites (sites 1 and 2) [132].

### 3.3. Anti-Inflammatory, Immunomodulatory, and Anticomplement Activities

Jin et al. have studied different factors that may affect the anticomplement activity of G-fucoidans. Among them were extraction methods, molecular weight, fucose:galactose molar ratio, sulfate content, uronic acid, type of glycosidic linkage, branching, and monomeric composition. The study concluded that larger molecular weights were more related to better activities [81]. G-fucoidans might also represent a novel and safer treatment strategy for chronic inflammation or related ailments. Six brown algal species have shown promising anti-inflammatory effects. Galactofucans from *Sargassum wightii* showed superior activity to aspirin, with EC_90_ values ranging from 0.2 to 1.22 mg/mL for inhibition of inflammatory-related enzymes [92,93]. Only the galactofucans from *Saccharina japonica* and *Lobophora variegata* have been tested in vivo with positive results [42,65,70,104]. Chen et al. showed that the investigated galactofucans from *S. japonica* were non-cytotoxic in the range of 3.125 to 25 μg/mL [65]. The anti-inflammatory was investigated in the form of fucoidan-based cream using fucoidan derived from *F. vesiculosus* of fucose:galactose ratio 1.0:0.05. A carrageenan-induced edema model in rats was employed, and the results showed 51–58% inhibition at 50 mg/kg fucoidan, which was comparable to the diclofenac effect. Such an effect was supposed to be linked with inhibition of IL-1*β*-induced COX-2 expression [133].

The reduction in the generation of nitric oxide (NO) and prostaglandin E2 (PGE2) via the downregulation of inducible nitric oxide synthase (iNOS) and cyclooxygenase-2 (COX-2) as well as the suppression of pro-inflammatory cytokines tumor necrosis factor (TNF)-α and interleukin (IL)-1*β* production via nuclear factor-kappa B (NF-κB) and mitogen-activated protein kinase (MAPK) have been pointed out as the mechanisms behind the anti-inflammatory activity reported for *S. japonica*. The fraction (LJNF3) could inhibit the production of 39.7% and 47.08% for TNF-α and IL-1β at 25 μg/mL [65,104]. This species presents a sulfated galactofucan that can be feasibly produced on a large scale due to its low-cost processing and superior anti-inflammatory activity [104]. Interestingly, the reviewed studies on *S. japonica* have been performed only on cultivated samples, meaning that seaweed cultivars can be good raw materials for anti-inflammatory compounds.

Furthermore, immunomodulatory compounds help to regulate immune function by accelerating or decelerating precise parts of the host response [134]. The complement system, an essential part of innate immunity, plays a pivotal role in eliminating “harmful” substances from the body. However, in some situations, its overactivity leads to diseases such as cancer or heart disease [82]. The immunomodulatory and anticomplement properties of galactofucans have been explored recently. Galactofucans from *Saccharina japonica* and *Lobophora variegata* exhibited immune-modulatory effects on RAW 264.7 cells (monocyte/macrophage-like cells) [70,102]. Studies on *S. japonica* and *Sargassum fusiforme* suggested that sulfated galactofucans were the active components of the anticomplement activity, with IC_50_ values of 4.5 and 5.5 µg/mL, respectively [81]. In addition, G-fucoidans from *S. hemiphyllum* presented higher anticomplement properties than sulfated galacto-fuco-xyloglucuronomannan from the same species [82]. All in all, galactofucans might be good candidates for immunomodulatory and anticomplement drugs.

### 3.4. Anticoagulant and Antithrombotic Activities

Fucoidans are well-known for their anticoagulant and antithrombotic activities. These polysaccharides have attracted extensive interest in discovering safer anticoagulants, with less hemorrhagic risk and good antithrombotic activity [135]. As part of this complex class of molecules, G-fucoidans also represent a source of potential antithrombotic drugs. For example, a sulfated galactofucan from *Spatoglossum schroederi* was 2-fold more potent than heparin in stimulating the synthesis of antithrombotic heparan sulfate by endothelial cells of rabbit aorta. In vivo experiments were key to clarifying the antithrombotic activity of this galactofucan, which initially did not show an anticoagulant effect during in vitro experiments. Such an effect was demonstrated for the fraction C at 100 µg/mL with an MW of 24 kDa [73]. Fucoidans can also enhance the plasma level of recombinant tissue plasminogen activator (rtPA), a protein commonly used as a non-interventional treatment to recanalize vessels occluded by acute thrombosis.

Moreover, a galactofucan from *U. pinnatifida* (specifically from *mekabu*) showed thrombolytic activity in vivo. This G-fucan’s competitive binding in vitro with PA inhibitor (PAI-1), a molecule that quickly neutralizes and inhibits rtPA, was the mechanism underlying fucoidan-mediated thrombolysis. It is worth mentioning that galactofucan from Korean samples showed better thrombolytic activity and binding affinity with PAI-1 than that from Russian samples. The authors suggested that Korean *mekabu* seemed to synthesize more active galactofucan than its Russian analog [114].

Furthermore, the higher percentage of galactose may also result in higher anticoagulant activity. For instance, Zayed et al. showed that fraction 6 (9% galactose) produced by dye affinity chromatography from *F. vesiculosus* exhibited a longer coagulation time (thrombin time 66 s) compared to other fractions, i.e., fractions 1 (7.4% galactose) and M (7.5% galactose) with 47 s and 31 s, respectively, at a fucoidan concentration of 10 µg/mL [8].

### 3.5. Antioxidant Activity

The scavenging effect of fucoidans on harmful oxidants, such as superoxide anion, hydrogen peroxide, hydroxyl radicals, and singlet oxygen, has attracted considerable interest from the food and pharmaceutical industries [136]. In this regard, galactofucan from the Tunisian brown seaweed *Cystoseira compressa* exhibited valuable antioxidant properties when subjected to various antioxidant tests, i.e., ferrous ion chelation, ferric ion reduction, and DPPH radical scavenging assays (Table 4). For instance, the DPPH assay resulted in an IC_50_ value of 430 μg/mL compared to 560 μg/mL for sodium alginate isolated from the same organism [64].

Similarly, a galactofucan from *Sargassum thunbergii* showed a higher scavenging effect of superoxide radical compared to vitamin C (ascorbic acid) [90] (Table 4). However, care should be considered when examining antioxidant activities, since contaminants such as co-extracted secondary metabolites (e.g., phlorotannins), not the galactofucans themselves, might be responsible for the reported bioactivities [1,107].

### 3.6. Other Biological Activities

Two recent studies have reported that galactofucans from *Sargassum siliquosum* exhibited antilipogenesis properties. According to the authors, the purified G-fucoidans (80 μg/mL) from this species induced a 28.9% reduction in lipid synthesis in human hepatoma cell line HepG2 after being induced by lipid accumulation with 1.0 mM oleate. The study used pioglitazone as a positive control at a concentration of 40 μg/mL [10,89]. In addition, the hypolipidemic effect was reported for a sulfated galactofucan from *Saccharina japonica* via inhibition of pancreatic lipase activity in a dose-dependent manner. Interestingly, this polysaccharide was not degraded by the human digestive system, likely due to its high molecular weight. Hence, this study might correlate such bioactivity not to the systemic effect, but through modulation of the microbiota composition. These results suggested that galactofucans could serve as fat-reducing health supplements without affecting the total sugar level [105].

In addition, in vitro and in vivo studies have shown antidiabetic and antihypertensive potentials of G-fucoidans from *Sargassum wightii*. In addition, galactofucans from this species showed superior antidiabetic activities compared to acarbose and diprotein-A (antidiabetic agents). The antidiabetic properties have proven to be significantly higher (*p* < 0.05) in terms of inhibitory activities for several enzymes involved in glucose metabolism, including *α*-amylase (IC_90_ = 0.9 mg/mL), *α*-glucosidase (IC_90_ = 1.4 mg/mL), and dipeptidyl peptidase-4 (IC_90_ = 0.1 mg/mL). In addition, the antihypertensive activity was tested against angiotensin-converting enzyme-I, showing an IC_90_ value of 0.2 mg/mL. Furthermore, these studies concluded that this compound was safe for consumption [92,93].

Fibroblast growth factor/fibroblast growth factor receptor (FGF/FGFR) signaling plays an essential role in various biological processes, including tumor growth and angiogenesis, regulation of cell chemotactic response process, cell proliferation, and differentiation [137,138]. Recently, it has been demonstrated that an LMW (10.9 kDa) galactofucan from *S. japonica* can regulate the FGFR-mediated MAPK signal pathway after incubation of BaF3 cells with 100 μg/mL, in comparison with heparin (2 μg/mL) [101]. Sulfated galactofucans might also represent a good regulator of FGF-1 when compared to the natural ligand, i.e., heparin [106].

In addition, a galactofucan from *Sargassum fusiforme* has shown promising activity on AD in vivo. During pharmacological experiments, this compound increased the cognitive abilities of scopolamine-, ethanol-, and sodium nitrite-treated mice against memory [80]. Furthermore, the radioprotective effect is a property recently attributed to fucoidans, including galactofucans from *S. feldmannii*. This species is the most promising source of radiosensitizing compounds among other *Sargassum* species at a concentration of 40 μg/mL, especially against human colon HT-29 and breast MDA-MB-231 cancer cells. It showed a significant, more than 30%, reduction in colony number of cancerous cells compared to irradiated cells [79,139].

Other properties reported for G-fucoidans, such as elastase inhibition and neuron protection activities, might be correlated to other well-studied activities (e.g., antitumor, antioxidant, or anti-inflammatory) [103,108]. Moreover, Pozharitskaya et al. used a G-fucoidan ioslated from *F. vesiculosus*, revealing its anti-hyperglycemic activity based on its inhibition of dipeptidyl peptidase-IV (DPP-IV) at IC_50_ 1.11 μg/mL [49].

## 4. Pharmacokinetic Studies

Despite the limited number of studies discussing the pharmacokinetics of galactofucans, including absorption, tissue distribution, metabolism, and excretion (ADME) behavior, pharmacokinetic study is an essential step for drug development, particularly after oral, topical, and intravenous administration. Such investigations have been included in recent studies and demonstrated highly promising results that qualified fucoidans to be potential candidates for further clinical trials in their pharmaceutical dosage forms [140,141,142].

G-fucoidan-based topical formulations, especially that derived from *F. vesiculosus*, have recently been employed in animal models such as rats. Following the administration of ointment containing 15% fucoidan at a dose of 50–150 mg/g, fucoidan was reported to be distributed into skin, striated muscle, and plasma with the highest concentration in striated muscle (AUC_0–48_= 2.2 μg·h/g) and without accumulation in plasma during five days of administration [142]. However, there was a literature conflict regarding fucoidan absorption from the digestive tract after oral intake due to its high molecular weight and subsequent mechanism of action, either via a systematic or modulation of gut microbiota composition effect [105,143]. The ELISA competitive antibody assay for sulfated polysaccharide showed low human plasma concentration of G-fucoidans following oral ingestion of 3 g/day for 12 days, where only 4.0 and 12.9 mg/L were resulted from oral ingestion of *U. pinnatifida*, equivalent to 10% and 75% pure fucoidan, respectively [143]. In addition, it was reported that fucoidan from *F. vesiculosus* following intragastric administration to the rats was found to be distributed to different organs such as the kidney, spleen, and liver. Interestingly, the kidney showed the maximum concentration, represented by AUC_0–t_= 10.7 µg·h/g and C_max_ = 1.2 µg/g after 5 h. In addition, it demonstrated a long absorption time and half-life time with a mean residence time of 6.8 h [140].

## 5. Conclusions and Future Perspectives

It has been well documented that fucoidans’ bioactivities are affected by four major factors, namely monomeric composition, glycosidic linkages, sulfate ester content, and sulfation pattern. Nevertheless, G-fucoidans or sulfated galactofucans are a unique class of fucoidans chemically and pharmacologically. Several brown seaweed species are recognized as good biogenic resources. They have attracted great attention in the last few years, especially following the great advances in marine biotechnology, chromatography, and spectroscopic techniques. Such advances could allow investigating the heterogeneous chemical composition of fucoidans and confirming the purity of isolated fucoidans. In comparison with other chemical classes of fucoidans, G-fucoidans’ chemical diversity has been reported mostly to be accompanied by various and potential pharmacological bioactivities, including antitumor, antiviral, and anticoagulant effects, especially those derived from *U. pinnatifida*. Previous literature has related some bioactivities with high fucose and sulfate contents or low molecular weight. Yet, the structure–activity relationships and the presence of galactose in higher percentages in the G-fucoidan chemical skeleton have not been revealed clearly. Nevertheless, the authors can assume that the superior bioactivities of G-fucoidans may be attributed to the branched chemical bones since galactose is always found in side chains, which is in agreement with previous literature showing that branched-chain fucoidans always exhibited more promising pharmacological effects. Hence, future studies should address these dark areas of G-fucoidans which can explain the secrets behind their outstanding biological effects. In addition, the seasonal variation and structural differences regarding G-fucoidans should be addressed in relation to bioactivities. The previous investigations on G-fucoidans have also proved their safety for consumption; therefore, G-fucoidans could be developed as a novel functional ingredient in the pharmaceutical and food industries. Furthermore, pharmacokinetic investigations for G-fucoidans should be further specified using different sources and formulations. Such research may help further clinical trials of this outstanding class of fucoidans in different pharmaceutical dosage forms.

## Figures and Tables

**Table 1:** Marine species of brown macroalgae (Phaeophyceae) producing G-fucoidans highlighting various structural features.

Brown Algae (Seaweed) Species	Source of Seaweed Biomass	Structural Characteristics						References
		Monosaccharide Composition	Glycosidic Bonds of Backbone	Molecular Weight (kDa)	Fucose/Galactose Ratio	Sulfate Content (%)	Sulfation Pattern	
**Dictyotales**								
*Canistrocarpus cervicornis*	Wild	Gal, fuc, glcAc, xyl,	ND		2	16.5	ND	[67]
*Dictyota dichotoma*	Wild	Gal, fuc, man, xyl, ara, rha, glc		23.6	1.5	33	ND	[68]
*D. implexa*	Wild	Gal, fuc	ND		1	18.3	ND	[69]
*Lobophora variegata*	Wild	Gal, fuc, Glc, man, xyl, glcAc; Gal, fuc; Gal, fuc, Glc	(1,3)- and (1,4)-α-l-fuc, and (1,3)-β-d-gal	35; ND; 1400	0.79; 0.5; 0.5	32.6; 0.2 *;15	At C4 (fuc)	[42,70]
*L. variegata*	ND	Gal, fuc	ND	360–1600	0.3	23.3–35.5	ND	[71]
*Padina boryana*	Wild	Gal, fuc	(1,4)-α-l-fuc, and (1,3)-β-d-gal	317.5/8.5	1.1	18.6	At C2 and C4 (fuc and gal)	[72]
*Spatoglossum schroederi*	Wild	Gal, fuc, xyl, glcAc; Gal, fuc, xyl;	(1,4)-*β*-d-gal, (1,4)-*α*-l-fuc, and (1,4)-*β*-d-xyl	21.5; 21.5–24	0.5; 0.5	19; 2.1–2.9 *	At C3 (gal) and C4 (fuc)	[73,74,75,76]
**Ectocarpales**								
*Adenocystis utricularis*	Wild	Gal, fuc, rha, man; Gal, fuc, rha; Gal, fuc, man	(1,3)-*α*-l-fuc	>100	5.53; 4.82; 5.53	23; 24; 23	At C4 (fuc and gal)	[48,77]
*Scytosiphon lomentaria*	Wild	Gal, fuc, rha, xyl, man, uronic acid	(1,3)-*α*-l-fuc, and (1,6)-*β*-d-gal	8.5	7.33	29.5	At C3 and C4 (fuc), and C3 (gal)	[78]
**Fucales**								
*Cystoseira compressa*	Wild	Gal, fuc	(1,3)- and (1,4)-*α*-l-fuc	100	2.32	14.7	At C2 and C4 (fuc)	[64]
*Sargassum duplicatum*	Wild	Gal, fuc	(1,4)-α-l-fuc and *β*-d-gal (alternating)	34–191	1	31.7	ND	[14]
*S. feldmannii*	Wild	Gal, fuc	(1,3)-*α*-l-fuc	183–184	2–2.6	25.3–32	At C2, C3 and C4 (fuc), and C2, C3, C4 and C6 (gal)	[14,79]
*S. fusiforme*	Wild	Gal, fuc, xyl, Glc, glcAc, man, uronic acid; Gal, fuc, xyl, man, rha, glcAc, Glc	(1,3)- and (1,4)-*α*-l-fuc	90; 118.3/3.9	2; 3.7	17.5; 28.5	At C3 (fuc)	[80,81]
*S. hemiphyllum*	Wild	Gal, fuc	(1,6)-*β*-d-gal, (1,3)- and (1,4)-*α*-l-fuc, and (1,3)-β-d-gal	148	4.5	32	At C2 and C4 (fuc)	[82]
*S. mcclurei*	Wild	Gal, fuc; Gal, fuc, man, xyl, glc	(1,3)-*α*-l-fuc	ND	1.4; 2	35; 30.5	At C2 and C4 (fuc)	[83,84]
*S. patens*	Wild	Gal, fuc, man, xyl, Glc, galactosamine	ND	424	1.9	14.4	ND	[85,86,87]
*S. polycystum*	Wild	Gal, fuc, glc; Gal, fuc, man, xyl, glc	(1,3)-*α*-l-fuc, and (1,6)-*β*-d-gal	39.5; ND	5.84; 1.48	33.6; 23.4	At C2 and C4 (fuc)	[84,88]
*S. siliquosum*	Wild	Gal, fuc, glc, xyl, man, rha; Gal, fuc, Glc, xyl, man, rha, uronic acid	(1,3)- and (1,4)-*α*-l-fuc	107.3; ND	1.9; 1.9	19.5; 20	At C4 and C6 (gal)	[10,89]
*S. thunbergii*	Wild	Gal, fuc	(1,3)-*α*-l-fuc	7.2–333.5	5.26–5.88	27.2–30.1	At C2 and C4 (fuc), and C4 (gal)	[46,63]
*S. thunbergii*	Purchased from local store	Gal, fuc	(1,4)-*α*-d-gal, and (1,3)-*β*-l-fuc	373	1.2	ND	NA	[90]
*S. wightii*	Wild	Gal, fuc, Glc, man; Gal, fuc	(1,3)-*α*-l-fuc	>3.5; ND	0.6; 3–3.5	379.1 ^†^; 8.1–19.5	At C2 and/or C4 (fuc), or C2 and C3 (gal)	[91,92,93]
*Turbinaria ornata*	Wild	Gal, fuc; Gal, fuc, man, xyl, glc	(1,3)-*α*-l-fuc	ND	5; 1.2	32; 25.6	At C2 and/or C4 (fuc), and/or C2, C3, C4/C6 (gal)	[84,94]
**Laminariales**								
*Alaria angusta*	Wild	Gal, fuc	(1,3)-*α*-l-fuc	ND	1.1	24	At C2 (fuc), and C2 and C4 (gal)	[95]
*Costaria costata*	Wild	Gal, fuc, man, rha, xyl	ND	ND	1.2	18.9	ND	[96]
*Ecklonia cava*	Wild	Gal, fuc, man, rha; Gal, fuc, rha, glc	ND	ND	4.8; 3.6	19.1; 22.2	At C2 (fuc)	[96]
*Laminaria hyperborea*	ND	Gal, fuc	(1,3)-*α*-l-fuc	469	44.5	53.8	At C2 and C4 (fuc)	[12]
*Saccharina angustata*	Wild	Gal, fuc, xyl, uronic acid	(1,3)-, (1,4) and (1,2)-*α*-l-fuc	56	9.1	4.2	At C4 (fuc and gal)	[97]
*S. gurjanovae*	Wild	Gal, fuc	(1,3)-*α*-l-fuc	123	3.2	25.1	At C2 and C4 (fuc), and C2 and/or C3 (gal)	[98]
*S. japonica*	Wild	Gal, fuc; Gal, fuc, man, xyl; Gal, fuc, man, rham, xyl; Gal, fuc, uronic acid, man, glcAc; Gal, fuc, Glc, man, rha, xyl; Gal, fuc, xyl, Glc, glcAc, rha, uronic acid	(1,3)-*α*-l-fuc	195/13.7; 1800; ND; 106.3; 23.5; 11	3.6; 1.1; 1.8; 9.1; 0.5; 10	21; 23.3; 23; 36.9; 18; 41.3	At C2 and C2/C4 (fuc)	[81,99,100,101,102]
*S. japonica*	Cultivated	Gal, fuc; Gal, fuc, man, rham, xyl, Glc; Gal, fuc, man, Glc, rha, xyl, uronic acid	(1,3)- and (1,4)-*α*-l-fuc	261.7; 131.5; 8.1	3.8; 2.1; 5.8	11.4; 9.1; 41.8	At C4 (fuc)	[65,103,104]
*S. japonica*	Provided by Fujian Yida Food Co.	Gal, fuc, man	ND	527.3	0.9	26.7	ND	[105]
*S. japonica*	ND	Gal, fuc	(1,3)-*α*-l-fuc, and (1,6)-*β*-d-gal	>10	3.5	48.3	At C4 and/or C2/C4 (fuc), and C4 and/or C3/C4 (gal)	[106]
*S. latissima*	Wild	Gal, fuc; Gal, fuc, xyl, man, Glc	(1,3)-*α*-l-fuc	416–449; 453	7.8; 4.1	0.8 ^‡^; 0.6 ^‡^	ND	[107,108]
*S. longicruris*	Wild	Gal, fuc, xyl, man, Glc, glcAc; Gal, fuc, xyl, man, Glc, galAc, glcAc		1529; 638	0.8; 0.4	17.6; 19.1	At C4 (fuc), and C3 (gal)	[109]
*Undaria pinnatifida*	Wild	Gal, fuc, man; Gal, fuc, rha; Gal, fuc, Glc, man, rha, xyl, ara	(1,3)- or (1,4)-*α*-l-fuc	ND; 290; ND	1.1; 1.2; 1.3	29; 0.94 ^‡^; ND	At C2, C3, C4 (fuc), or C2 and C4 (fuc and/or gal)	[99,110,111]
*U. pinnatifida* (sporophylls)	Wild	Gal, fuc, xyl, man	(1,3)-*α*-l-fuc	>150	1.5	15	ND	[112,113]
	Cultivated	Gal, fuc; Gal, fuc, man; Gal, fuc, xyl, man; Gal, fuc, man, xyl, uronic acid	(1,3)-*α*-l-fuc, and (1,3)-, (1,4)-, (1,6)-*β*-d-gal	ND; 1.4–3.7; 1246; 2100	1.4; 1.1; 1.1; 5	31; 8.4; 9.2; 7.4	At C2/C4 (fuc), and C3/C6 (gal)	[114,115,116]
	From mussel farms	Gal, fuc, xyl, Glc, man; Gal, fuc, xyl, Glc, man, uronic acid		171; >150	1.5; 1.5	15; 15	ND	[44,117]
*U. pinnatifida*	From Marine Resources Pty Ltd.			ND	ND	ND	ND	[118]
	From Marinova Pty Ltd.	Gal, fuc, xyl, man	(1,3)-*α*-l-fuc	51.7	1.3	21.5	At C2 and C4 (fuc)	[119]
	ND			ND	ND	ND	ND	[120]
*U. pinnatifida* (sporophylls)	ND	Gal, fuc; Gal, fuc, uronic acid; Gal, fuc, xyl, man	(1,3)-*α*-l-fuc, and (1,3)-, (1,4)-, (1,6)-*β*-d-gal	9; 9; 104.4	0.9; 0.9; ND	10.4; 10.4; 21	At C2 (fuc), and C3 and C6 (gal)	[121,122,123]
**Sphacelariales**								
*Sphacelaria indica*	Wild	Gal, fuc, xyl, man, Glc	(1,3)-*α*-l-fuc	26	3.3	4	At C4 (fuc)	[124]

ND, not detailed; NA, not applicable; * reported as molar ratio to fucose; ^†^ reported as mg/g fucoidan; ^‡^ reported as degree of sulfation.

**Table 2:** G-fucoidans showing anticancer/antitumor activity with their respective sources and half-maximal inhibitory concentrations (IC_50_). Comparisons with standard or commercial compounds are also shown.

Source	IC_50_	Compared with Standard/Commercial Compounds?	References
*Saccharina latissima*	0.35 µg/mL (elastase inhibition)	Yes. Superior to commercial heparins (UFH and tinzaparin)	[107]
*Sargassum polycystum*	84.63 µg/mL (leukemia cells) and 93.62 µg/mL (breast cancer cells)	No	[84,88]
*S. thunbergii*	29.7–93.5 μg/mL (inhibition of FGF1 binding) and 4.0–6.8 μg/mL (inhibition of FGF7 binding)	No	[46,63]
*Undaria pinnatifida* (sporophylls)	0.10 mg/mL (breast adenocarcinoma) and 0.15 mg/mL (lung carcinoma)	Yes. Superior to commercial fucoidan from *Fucus* for both cancer cell lines	[44,117,130]

**Table 3:** Summarized antiviral activity of G-fucoidans with their respective sources and half-maximal effective or inhibitory concentrations (EC_50_/IC_50_). Comparisons with antiviral drugs are also shown.

Source	EC_50_/IC_50_	Compared with Antiviral Drugs?	References
*Adenocystis utricularis*	0.6–0.9 µg/mL (HIV-1)	Yes. Superior to azidothymidine	[48,77]
	0.3 µg/mL (HSV-1) and 0.5 µg/mL (HSV-2)	No	[48]
*Dictyota dichotoma*	7.5 µg/mL (HSV-1), and 15.6 µg/mL (CVB3)	Yes. Superior to ribavirin	[68]
*Saccharina japonica*	0.001–0.005 µg/mL (HIV-1)	No	[100]
	0.2–25 µg/mL (HSV-1)	Yes. Inferior to acyclovir and similar to heparin	[97]
*Sargassum mcclurei*	0.96 µg/mL (HIV-1)	Yes. Inferior to AMD3100 (plerixafor)	[84]
*S. patens*	1.3 µg/mL (HSV-2), 5.5 µg/mL (HSV-1), and 4.1 µg/mL (HSV-1 acyclovir-resistant strain)	No	[85,86,87]
	>50 µg/mL (virucidal activity against HSV-2), 1.3–1.65 µg/mL (plaque formation), 1.85–3.5 µg/mL (inhibition of virus adsorption)	No	
	1.5–5.5 mg/mL (HSV-1 replication) and 3–4 mg/mL (HSV-1 adsorption)	Yes. Similar to acyclovir	
*S. polycystum*	0.34 µg/mL (HIV-1)	Yes. Inferior to AMD3100 (plerixafor)	[84]
*Scytosiphon lomentaria*	0.76 µg/mL (HSV-1) and 1.34 µg/mL (HSV-2)	No	[78]
*Sphacelaria indica*	1.3 µg/mL (HSV-1)	Yes. Superior to acyclovir when added to the overlay medium after penetration of the viruses into the host cell	[124]
*Turbinaria ornata*	0.39 µg/mL (HIV-1)	Yes. Inferior to AMD3100 (plerixafor)	[84]
*Undaria pinnatifida*	0.77 µg/mL (HSV-1)	Yes. Superior to acyclovir	[111]
	32 µg/mL (HSV-1) and 0.5 µg/mL (HSV-2)	Yes. Superior to acyclovir	[120]
*U. pinnatifida* (sporophylls)	2.5 µg/mL (HSV-1), 2.6 µg/mL (HSV-2), and 1.5 µg/mL (HCMV)	No	[121,122,123]
*U. pinnatifida*	1.1 µg/mL (HSV-1), 0.1 µg/mL (HSV-2), and 0.5 µg/mL (HCMV)	No	[99,110,111]
	3.1 µg/mL (HSV-1) and 1.6 µg/mL (HSV-2)	No	[118]

**Table 4:** G-fucoidans showing antioxidant activity with their respective sources and half-maximal effective or inhibitory concentrations (EC_50_/IC_50_). Comparisons with standard or commercial compounds are also shown.

Source	EC_50_/IC_50_	Compared with Standard/Commercial Compounds?	References
*Cystoseira compressa*	0.43 mg/mL (DPPH)	Yes. Inferior to ascorbic acid and butylated hydroxyanisole	[64]
*Sargassum siliquosum*	2.58 mg/mL (DPPH)	No	[10]
*S. thunbergii*	0.22 mg/mL (superoxide radical), and 0.88 mg/mL (hydroxyl radical)	Yes. Similar (hydroxy radical) or superior (superoxide radical) to vitamin C	[90]
